# Human follicular fluid proteome reveals association between overweight status and oocyte maturation abnormality

**DOI:** 10.1186/s12014-020-09286-7

**Published:** 2020-06-08

**Authors:** Xin Liu, Yanhua Wang, Peng Zhu, Jiahui Wang, Juan Liu, Ning Li, Wenting Wang, Wendi Zhang, Chengli Zhang, Yanwei Wang, Xiaofang Shen, Fujun Liu

**Affiliations:** 1 Central Laboratory, The Affiliated Yantai Yuhuangding Hospital of Qingdao University, Yantai, 264000 Shandong People’s Republic of China; 2 Department of Medical Records Room, Weifang People’s Hospital, Weifang, 261041 Shandong People’s Republic of China; 3 Reproductive Center, Beijing BaoDao Obstetrics and Gynecology Hospital, Beijing, 100000 People’s Republic of China

**Keywords:** Human follicular fluid, Overweight status women, In vitro fertilization, iTRAQ, Oocyte maturation

## Abstract

**Background:**

Human follicular fluid (HFF), which is composed by essential proteins required for the follicle development, provides an important microenvironment for oocyte maturation. Recently, overweight status has been considered as a detrimental impact factor on oocyte maturation, but whether HFF proteome could provide protein markers for assessing overweight-based oocyte maturation deficiency is still unknown.

**Methods:**

To reveal the HFF-based molecular characteristics associated with abnormal oocyte maturation, an iTRAQ-based comparative proteomic analysis was performed to investigate different HFF protein expression profiles from normal weight women and overweight status women.

**Results:**

Two hundred HFF proteins were quantified in our data, of which 43% have not been overlapped by two previous publications. Compared with the HFF proteins of normal weight women, 22 up-regulated HFF proteins and 21 down-regulated HFF proteins were found in the overweight status women. PANTHER database showed these altered HFF proteins participated in development, metabolism, immunity, and coagulation, and STRING database demonstrated their complicated interaction networks. The confidence of proteomic outcome was verified by Western blot analysis of WAP four-disulfide core domain protein 2 (WFDC2), lactotransferrin (LTF), prostate-specific antigen (KLK3), fibronectin (FN1), and glyceraldehyde 3-phosphate dehydrogenase (GAPDH). Further, ELISA assay indicated WFDC2 might be a potentially useful candidate HFF marker for the diagnosis of oocyte maturation arrest caused by overweight status.

**Conclusions:**

Our work provided a new complementary high-confidence HFF dataset involved in oocyte maturation, and these altered HFF proteins might have clinical relevance and diagnostic and prognostic value for abnormal oocyte maturation in overweight status women.

## Introduction

Infertility remains a rising worldwide problem, characterized by the failure to achieving clinical pregnancy after 12 months of regular attempts of natural fertilization. About 9% of reproductive-aged couples (186 million people) suffer from childlessness, of which male and female factors approximately contribute equally to infertility [1]. Unfortunately, in some developing countries, infertility is still a massive social burden for women due to limited access to the help of assisted reproductive technology (ARTs) [2–4]. Lots of factors are associated with female infertility, such as polycystic ovary syndrome (PCOS), endometriosis, anovulation, and tubal, uterine, endocrine, paracrine, and unexplained factors. Recently, overweight status has been considered to have a detrimental impact on female reproductive health, which involves disorder of the ‘hypothalamic-pituitary-ovarian axis’. Consequently, these dysfunctional female gonads negatively influence oocyte development and hormone secretion, leading to ovulatory dysfunction and female infertility [5, 6].

Human follicular fluid (HFF) provides a special and ideal microenvironment for oocyte maturation, composed of steroid hormones, metabolites and proteins, which are required for follicle development and oocyte maturation [7–9]. Importantly, HFF can be easily obtained during the aspiration of oocytes from follicle in the procedure of in vitro fertilization (IVF), which facilitates it to be as a useful source for the biomarker screening for oocyte maturation [10]. Proteomic technology has been applied to explore key molecules for the better understanding of the physiological processes involved in follicle development and oocyte maturation. Two-dimensional gel electrophoresis has been carried out to explore the differential HFF proteins between younger and older women. As a result, some HFF proteins including complement C3, C4, and serotransferrin showed significant changes in the aged group [11]. Ambekar et al. has applied SDS-PAGE, OFFGEL fractionation, and SCX-based approach combined with LC–MS/MS to identify 480 HFF proteins for monitoring oocyte and embryo quality [12]. Lewandowska et al. further used a SWATH-MS methodology of ultrafiltration, optional immunodepletion, and high pH RP-HPLC separation coupled by spectral libraries to quantify 108 HFF proteins and 250 peptides in all tests [13].

Previously, we have adopted a proteomic approach of dual RP-HPLC combined with LC-MALDI TOF/TOF mass spectrometry to investigate the HFF protein profile. A total of 219 unique high-confidence HFF proteins (FDR < 0.01) were successfully identified, which were associated with various molecular functions, such as immunity, complement and coagulation cascade, and transportation, and participated in the implicated interaction networks [14]. Considering the overweight status as a risk factor for women infertility [15], we performed a retrospective cohort research in 411 non-PCOS women patients undergoing their first IVF-embryo transplantation (IVF-ET) treatment. The results indicated that overweight status negatively affected oocyte maturation [16].

Up to now, it is still unknown about the relationship between oocyte maturation arrest and the alteration of HFF protein composition in overweight status women. Therefore, in the present work, we further applied an integrative proteomic approach of iTRAQ labeling, dual RP-HPLC separation and MALDI mass spectrometry identification to deeply explore the differential HFF proteins related to oocyte maturation between the normal weight women and overweight status women undergoing IVF treatment. Therefore, this study will not only facilitate to discover the important proteins influencing the process of oocyte maturation, but also resolve the complicated interaction networks of these altered molecules, which will profile potential biomarkers for the screening of HFF proteins associated with oocyte maturation.

## Materials and methods

### Study subjects and sample collection

For the recruitment of female patients, the causes of infertility were simple tubal factor, age was no more than 38 years, the concentration of serum FSH was less than 12 mIU/mL, the ovulation stimulation underwent the long protocol, and the patients were treated with their first egg retrieval cycle. Exclusion criteria included endocrine diseases, polycystic ovary syndrome, endometriosis, receiving intracytoplasmic sperm injection-embryo transfer (ICSI-ET), and chromosomal abnormalities. Referring to the WHO criteria [17], the recruited patients were divided into the normal weight group (18.5 ≤ BMI ≤ 24.9 kg/m^2^) and the overweight status group (BMI ≥ 25 kg/m^2^) (Table 1). According to previous method [18], HFF samples were collected when the oocytes were aspirated under the guidance of trans-vaginal ultrasound. The resultant HFF samples were clear and without the contamination of the flushing medium. After centrifugation at 10,000×*g* for 30 min at 4 °C, the concentrations of cell debris-free HFF samples was determined by the Bradford method [19]. This work has been approved by the Ethics Committee of Beijing BaoDao Obstetrics and Gynecology Hospital, and written informed consents were obtained from all participants.

**Table 1:** Clinical characteristics of study participants

Couple	Characteristics	Normal weight	Overweight status	*p* value
Male partner	Age	33.5 ± 0.1	33.3 ± 0.2	0.334
	Body mass index (BMI) (kg/m^2^)	25.1 ± 0.4	24.6 ± 0.5	0.403
	Semen volume (ml)	3.4 ± 0.2	3.2 ± 0.2	0.556
	Sperm concentration (million sperm/ml semen)	90.3 ± 7.4	89.8 ± 4.7	0.95
	Sperm motility (% progressively motile sperm)	38.9 ± 1.6	39.3 ± 1.6	0.863
Female partner	Age	33.1 ± 0.1	32.9 ± 0.1	0.286
	Body mass index (kg/m^2^)	22.2 ± 0.2	28.4 ± 0.1	< 0.01
	Basal FSH levels (U/l)	5.5 ± 0.2	5.3 ± 0.2	0.568
	Basal LH levels (U/l)	4.6 ± 0.3	4.2 ± 0.5	0.331
	Basal E2 levels (U/l)	37.4 ± 3.7	37.7 ± 1.8	0.95
	Number of oocytes collected	11.0 ± 0.8	9.5 ± 0.6	0.14
	MII (n)	8.3 ± 0.7	6.0 ± 0.4	< 0.01

Results are expressed as mean ± SD for each parameter. Comparison was done by unpaired t test. *p* < 0.05 is statistically significant

### iTRAQ labeling of the HFF proteins from normal weight and overweight status women

According to the previous method [20], equal amounts of HFF samples from 20 normal weight women, and 20 overweight status women were pooled. The resultant pools from the normal weight and overweight status women were respectively divided into two tubes (100 μg protein each tube), and the pH was adjusted to 8.5. The pools were treated with 20 mM dithiothreitol (DTT) at 56 °C for 60 min, and 50 mM iodoacetamide (IAA) in the dark for 30 min. Consequently, the samples were digested by trypsin (sequencing grade, Promega, France) (W/W, 1:50 enzyme/protein) at 37 °C overnight. The resultant peptides were tagged by iTRAQ reagents, and then a SPD2010 SpeedVac concentrator system (Thermo, North Carolina, USA) was used to dry the labeling peptides for further analysis.

### First dimension high pH RP-HPLC

The first dimension RP-HPLC separation was carried out using a PF-2D RP-HPLC System (Beckman Coulter, Inc., Fullerton, CA, USA) with a Durashell RP column (5 µm, 150 Å, 250 mm × 4.6 mm i.d., Agela). A 65-min eluent gradient was developed by mobile phase A (2% acetonitrile, adjusted pH to 10.0 using NH_3_.H_2_0) and B (98% acetonitrile, adjusted pH to 10.0 using NH_3_.H_2_0) at a flow rate of 0.8 mL/min. A total of 20 eluent fractions were collected for the next fractionation.

### Second dimension low pH RP-HPLC combined with the determination of MALDI mass spectrometry

The above fractionations were dried by a SPD2010 SpeedVac concentrator system (Thermo, North Carolina, USA) and re-dissolved in 30 μL of buffer A (0.1% (v/v) formic acid, 2% (v/v) acetonitrile in water). The resultant peptides were eluted by a C18 AQ 150 × 0.2 mm column (3 μm, Michrom, USA) using a 90-min linear gradient formed by buffer A (2% acetonitrile, 0.1% formic acid) and buffer B (98% acetonitrile, 0.1% formic acid) at a flow rate of 0.5 μL/min. At the same time, each fraction was mixed with α-Cyano-4-hydroxycinnamic acid matrix solution (5 mg/mL in 70% acetonitrile, 0.1% trifluoroacetic acid) at a flow rate of 2 μL/min pushed by additional syringe pump and spotted on the MALDI plate using the Tempo™ LC-MALDI Spotting System (AB SCIEX, USA). As a result, 616 spots per fraction were spotted on a 123 × 81 mm LC-MALDI plate and determined by a MALDI-TOF/TOF 5800 mass spectrometer (AB SCIEX, USA). The full-scan m/z range of MS data acquisition was from 800 to 4000, and the top 40 ions were selected for the further analyzation by MS/MS.

### Identification and quantification of differential HFF proteins between the normal weight and overweight status women

The spectra of HFF proteins were analyzed by the ProteinPilot™ software (version 4.0.1, AB SCIEX) using a reviewed UniProtKB/Swiss-Prot database (20,316 sequences, 2018_02 released). Meanwhile, a decoy database (programmed in the ProteinPilot™ software) was applied to ensure the data quality (FDR < 0.01). The searching parameters were as following: iTRAQ 4plex mode, trypsin enzyme, maximum allowed missed cleavages 1, and carbamidomethyl cysteine. The HFF protein abundances were calculated by all quantified labeled peptides through the areas of the monoisotopic peaks. The statistical alteration of HFF proteins with a confidence interval of 99% (*p* value < 0.01) was determined by the average ratio of four pairs (116:114, 117:114, 116:115, and 117:115) in two repeat experiments.

### Bioinformatics analysis

The altered HFF proteins were classified by the online databases PANTHER (Protein ANalysis THrough Evolutionary Relationships) (released 13.1, 2018-02-03) (http://pantherdb.org/) and DAVID (The Database for Annotation, Visualization and Integrated Discovery) (released 6.8, 2016-10) (https://david.ncifcrf.gov/), and the literatures from PubMed (https://www.ncbi.nlm.nih.gov/pubmed). Each HFF protein was categorized into only one classification. The protein–protein interaction network was established by the STRING (search tool for recurring instances of neighboring genes) database (released 10.5, 2017-05-14).

### Western blot analysis

After separation of 50 μg protein of each HFF sample by a 12% SDS-PAGE gel, the resultant proteins were electronically transferred onto a nitrocellulose membrane. Further, 5% (w/v) skimmed milk was used to block the above membrane at 37 °C for 1 h, and the primary antibodies (WAP four-disulfide core domain protein 2 (WFDC2), ab109298, Abcam, Cambridge, USA; lactotransferrin (LTF), ab109000, Abcam, Cambridge, USA; Prostate-specific antigen (KLK3), ab76113, Abcam, Cambridge, USA; Fibronectin (FN1), ab32419, Abcam, Cambridge, USA; glyceraldehyde 3-phosphate dehydrogenase (GAPDH), 60004-1-Ig, Proteintech Group Chicago, USA) were respectively added for incubation at 4 °C overnight. The resultant membranes were washed with TBST for three times, and then incubated with horse-radish peroxidase-conjugated secondary antibody (diluted 1:5000, Zhong-Shan Biotechnology, Beijing, China) at room temperature for 1 h. The enhanced chemiluminescence detection reagents (Pierce, Rockford, IL, USA) was used to visualize the immunoreactive proteins.

### Validation by ELISA

Follicular Fluid proteins were further validated with HFF samples from 25 normal weight women and 25 overweight status women. Concentrations of WFDC2 in fivefold diluted HFF samples were measured by commercial ELISA kit (Li su (Shanghai) Biotechnology Co., Ltd, China) referring to the manufacture’s protocol. Protein concentrations were calculated by comparing the optical density of samples at 450 nm wavelength with the standard curve using a microplate spectrophotometer (Multiskan FC, Thermo scientific).

## Results

### Comparative analysis of HFF proteins between the normal weight and overweight status women

**Fig. 1:**
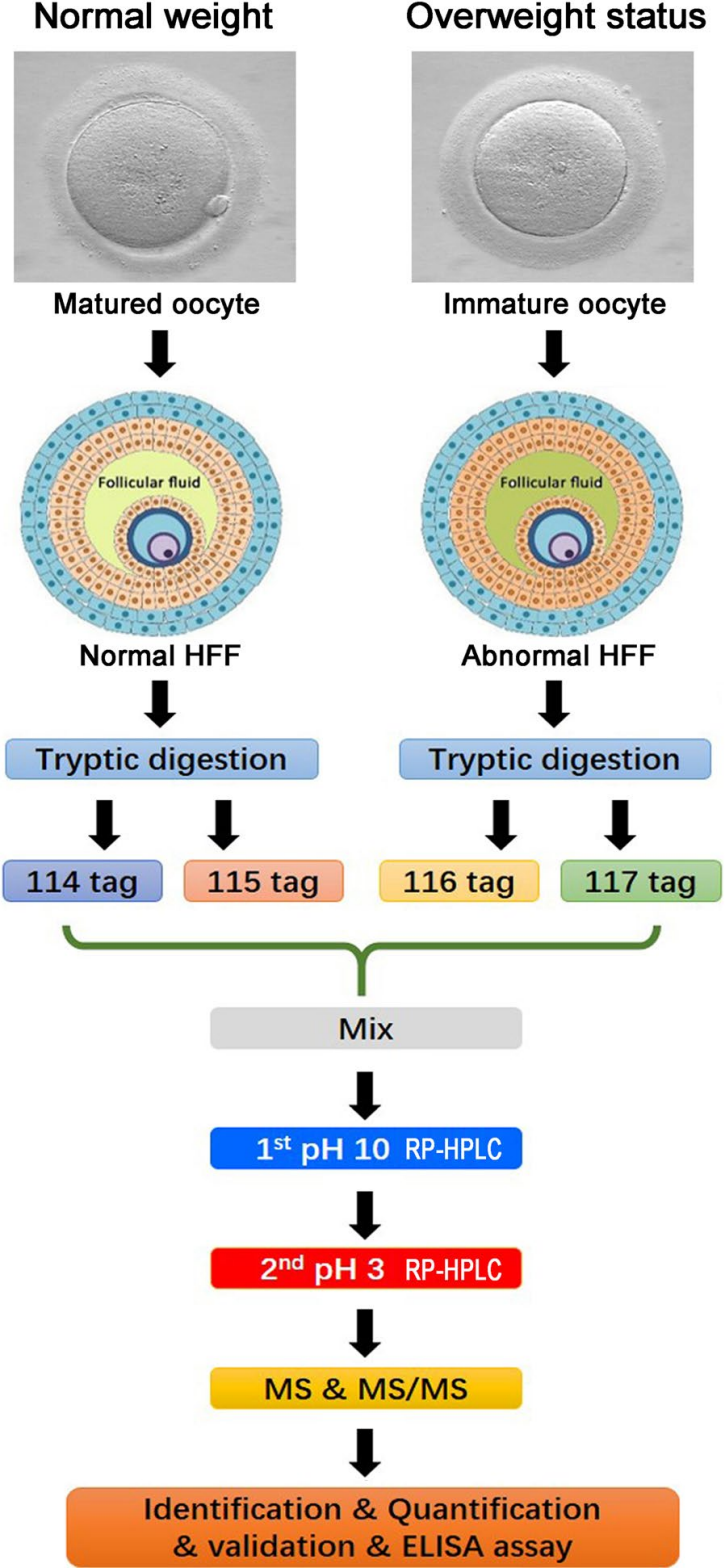
Experimental workflow of iTRAQ-based HFF proteomic study of the normal and the overweight status women. Equal amounts of HFF proteins from 20 normal-weight women and 20 overweight status women were pooled, digested by trypsin, and labelled by iTRAQ labels. After the labeled samples were separated by a pH 10.0 and a pH 3.0 reversed phase chromatography, the resultant fractions were spotted on the MALDI plates. Then, the peptides were sequenced by the 5800 MALDI-TOF/TOF mass spectrometry, and the quantification of HFF proteins were performed by the software of Protein pilot (version 4.0)

**Fig. 2:**
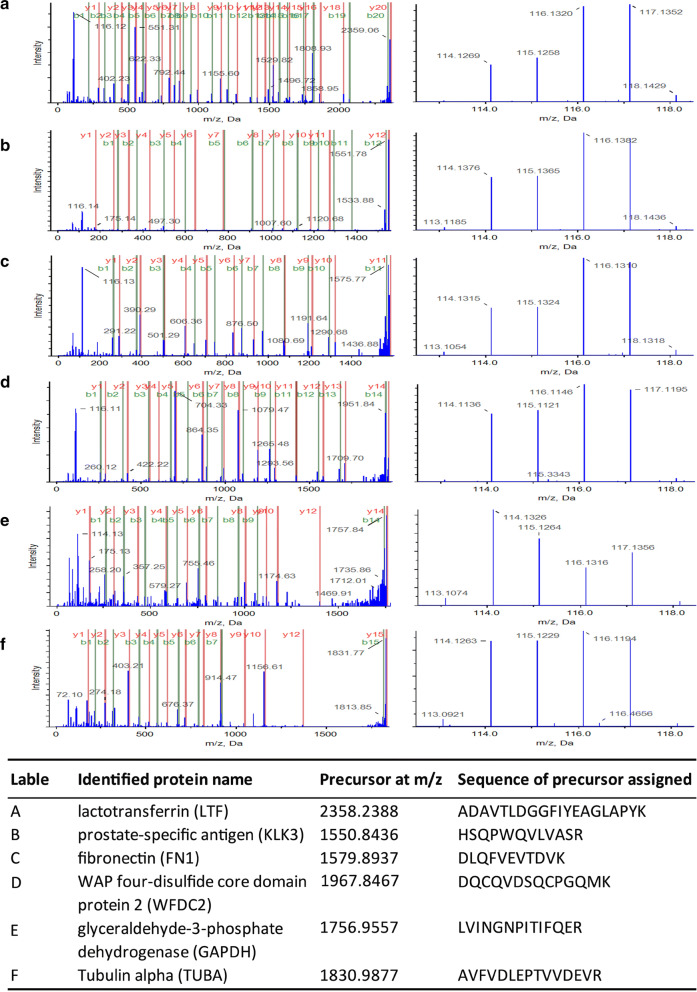
Representative spectrum of differential HFF protein sequenced by 5800 MALDI TOF/TOF mass spectrometry. The left column were MS/MS maps with protein identifications in the table blow. The right maps represented the ions labelled by iTRAQ reagent

Figure 1 indicated representative images of a matured oocyte and an immature oocyte, and the flowchart of iTRAQ-based proteomic analysis of HFF proteins from the normal weight and overweight status women. Totally, 200 HFF proteins were identified and quantified using the ProteinPilot™ software to search against the reviewed Swiss-Prot human database (20,316 sequences, 2018_02 released) with a high confidence (FDR < 0.01) (Additional file 1: Table S1). Compared with the HFF proteins of normal-weight women, 22 HFF proteins were up-regulated (ratio_overweight status/normal weight_ > 1.380, *p *< 0.01) (Table 2), and 21 HFF proteins were down-regulated (ratio_overweight status/normal weight_ < 0.658, *p *< 0.01) (Table 3) in the overweight status women. Figure 2 showed the representative MS/MS spectrum of differential iTRAQ-labelled peptide noted with most b-ions and y-ions.

**Table 2:** Higher abundant HFF proteins in overweight status women (n = 22)

Swiss-Prot accession number	Protein name	Ratio (Mean ± SD) overweight status/normal weight
P21926	CD9 antigen	2.104 ± 0.152
P02788	Lactotransferrin	2.018 ± 0.027
P01861	Immunoglobulin heavy constant gamma 4	1.833 ± 0.085
P07108	Acyl-CoA-binding protein	1.810 ± 0.042
P15309	Prostatic acid phosphatase	1.805 ± 0.059
P02751	Fibronectin	1.759 ± 0.043
P03973	Antileukoproteinase	1.747 ± 0.123
P07288	Prostate-specific antigen	1.715 ± 0.061
P08118	Beta-microseminoprotein	1.696 ± 0.073
Q16610	Extracellular matrix protein 1	1.617 ± 0.056
Q12841	Follistatin-related protein 1	1.584 ± 0.083
P12277	Creatine kinase B-type	1.572 ± 0.200
P02763	Alpha-1-acid glycoprotein 1	1.558 ± 0.062
Q14508	WAP four-disulfide core domain protein 2	1.555 ± 0.095
P54108	Cysteine-rich secretory protein 3	1.546 ± 0.060
P61916	Epididymal secretory protein E1	1.541 ± 0.039
P15144	Aminopeptidase N	1.540 ± 0.015
P13473	Lysosome-associated membrane glycoprotein 2	1.470 ± 0.110
O60635	Tetraspanin-1	1.464 ± 0.048
Q08380	Galectin-3-binding protein	1.460 ± 0.032
P35542	Serum amyloid A-4 protein	1.426 ± 0.066
P61769	Beta-2-microglobulin	1.421 ± 0.142

Annotations of altered proteins identified in HFF from the overweight status women compared with the normal weight women. Values > 1.380 (*p *< 0.01) correspond to higher abundance in the overweight status women group. Standard deviation (SD)

**Table 3:** Lower abundant HFF proteins in overweight status women (n = 21)

Swiss-Prot accession number	Protein name	Ratio (Mean ± SD) overweight status/normal weight
P01624	Immunoglobulin kappa variable 3–15	0.653 ± 0.095
A0A0A0MS15	Immunoglobulin heavy variable 3–49	0.646 ± 0.044
P02748	Complement component C9	0.646 ± 0.047
Q96PD5	*N*-acetylmuramoyl-l-alanine amidase	0.646 ± 0.071
P00338	l-lactate dehydrogenase A chain	0.632 ± 0.077
P04406	Glyceraldehyde-3-phosphate dehydrogenase	0.628 ± 0.184
P37802	Transgelin-2	0.626 ± 0.027
Q6IS14	Eukaryotic translation initiation factor 5A-1-like	0.624 ± 0.142
P18065	Insulin-like growth factor-binding protein 2	0.624 ± 0.176
A0A0J9YXX1	Immunoglobulin heavy variable 5-10-1	0.614 ± 0.034
Q99439	Calponin-2	0.605 ± 0.177
P15121	Aldose reductase	0.588 ± 0.113
P98160	Basement membrane-specific heparan sulfate proteoglycan core protein	0.581 ± 0.016
Q02383	Semenogelin-2	0.580 ± 0.009
P01036	Cystatin-S	0.563 ± 0.050
P01019	Angiotensinogen	0.531 ± 0.062
P00748	Coagulation factor XII	0.523 ± 0.036
P31948	Stress-induced-phosphoprotein 1	0.507 ± 0.045
P07195	l-Lactate dehydrogenase B chain	0.498 ± 0.070
P55072	Transitional endoplasmic reticulum ATPase	0.468 ± 0.100
Q9HC38	Glyoxalase domain-containing protein 4	0.442 ± 0.067

Annotations of altered proteins identified in HFF from the overweight status women compared with the normal weight women. Values < 0.658 (*p *< 0.01) correspond to lower abundance in the overweight status women group. Standard deviation (SD)

### Bioinformatics analysis of the HFF proteome

**Fig. 3:**
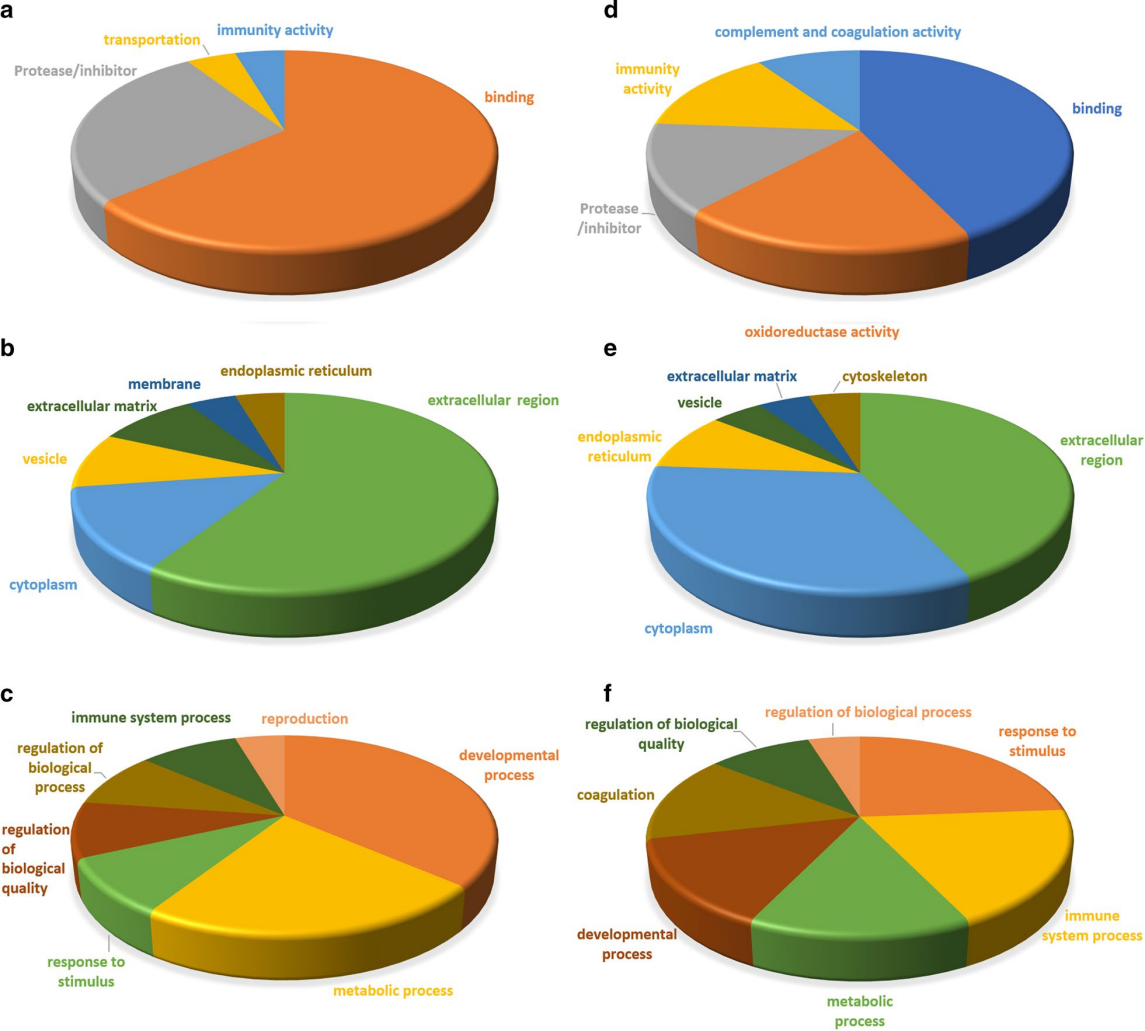
Pie diagrams of GO classifications of higher abundance HFF proteins (**a**–**c**) and lower abundance HFF proteins (**d**–**f**) in the overweight status women. **a**, **d** are molecular functions, **b**, **e** are subcellular localizations, and **c**, **f** are biological processes

Referring to the PANTHER, DAVID and PubMed databases, 43 altered HFF proteins were classified into GO categories (molecular function, subcellular localization, and biological process). Each HFF protein was place into only one classification (Fig. 3). In the molecular function category, the majority of the higher abundance HFF proteins in the overweight status women was related to binding function (Fig. 3a), whereas most lower abundance HFF proteins in the overweight status women were involved in binding and oxidoreductase activity (Fig. 3d). Regarding subcellular localization, the major higher abundance HFF proteins in the overweight status women were located in extracellular region (Fig. 3b), whereas the main locations of the lower abundance HFF proteins in the overweight status women were cytoplasm and extracellular region (Fig. 3e). Regarding biological process, the main higher abundance HFF proteins in the overweight status women was involved in developmental process and metabolic process (Fig. 3c), whereas the prevalent processes of the lower abundance HFF proteins in the overweight status women were response to stimulus and immune system process (Fig. 3f).

**Fig. 4:**
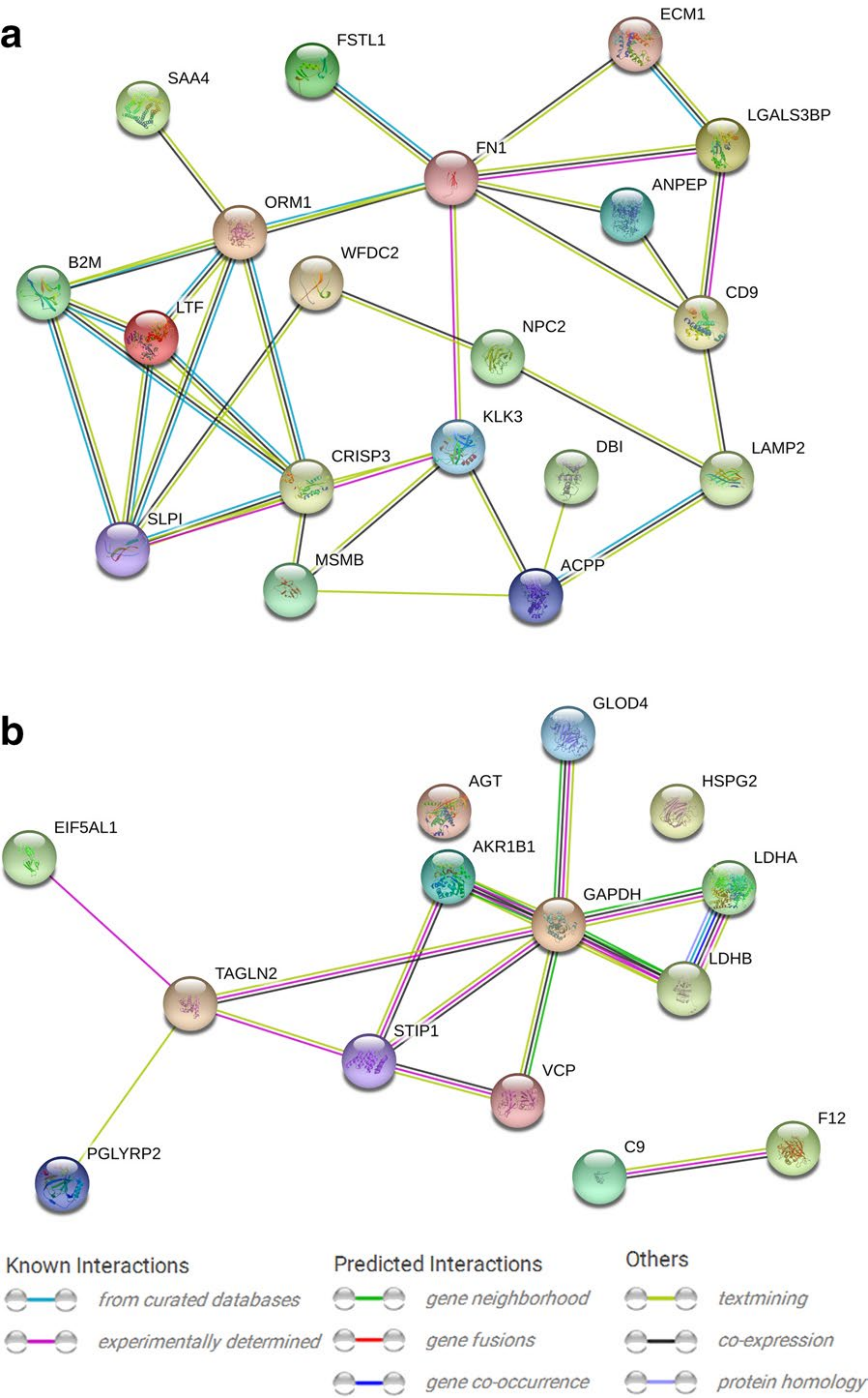
Representative protein–protein interaction networks of the higher abundance HFF proteins (**a**) and lower abundance HFF proteins (**b**) in the overweight status women. The relationships were derived from the curated databases, experimentally determined, gene neighborhood, gene fusions, gene co-occurrence, textmining, co-expression, and protein homology

A protein–protein interaction network was retrieved from the STRING database. Among the higher abundance HFF proteins of the overweight status women, 19 altered HFF proteins were in connection with other proteins, leading to 34 paired relationships (PPI enrichment *p* < 1.0e−16) (Fig. 4a), whereas among the lower abundance HFF proteins of the overweight status women, 12 altered HFF proteins were in connection with other proteins, leading to 15 paired relationships (PPI enrichment *p* = 0.000116) (Fig. 4b).

### Comparison of present HFF proteome and previous HFF proteomes

**Fig. 5:**
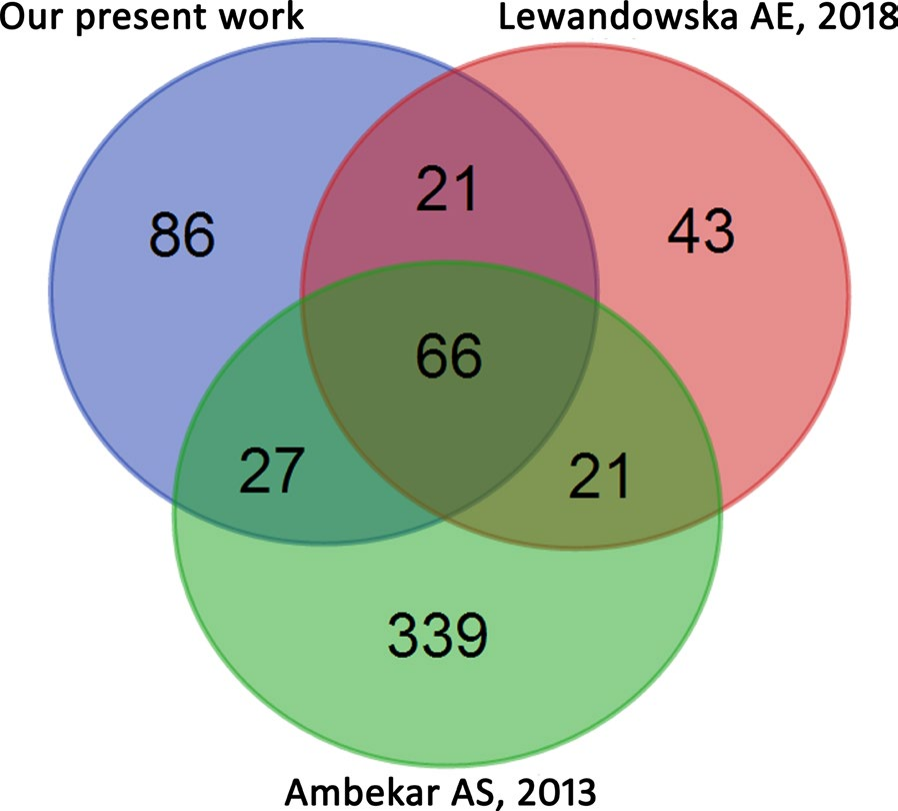
Venn diagram of the overlaps of the present work and the other two HFF proteomes 13, 18]

To display the overlap of the HFF proteomes among different research datasets, the previously reported HFF proteomic datasets with high data quality (FDR < 1%) [13, 14] were selected to compare with our HFF proteome (Fig. 5). The results indicated that 57% of HFF proteins in our data were included in either of the two previous HFF datasets, whereas only 33% of HFF proteins were common in all three HFF datasets. The overlap showed the complementarity among the differential labs.

### Western blotting analysis and ELISA assay

**Fig. 6:**
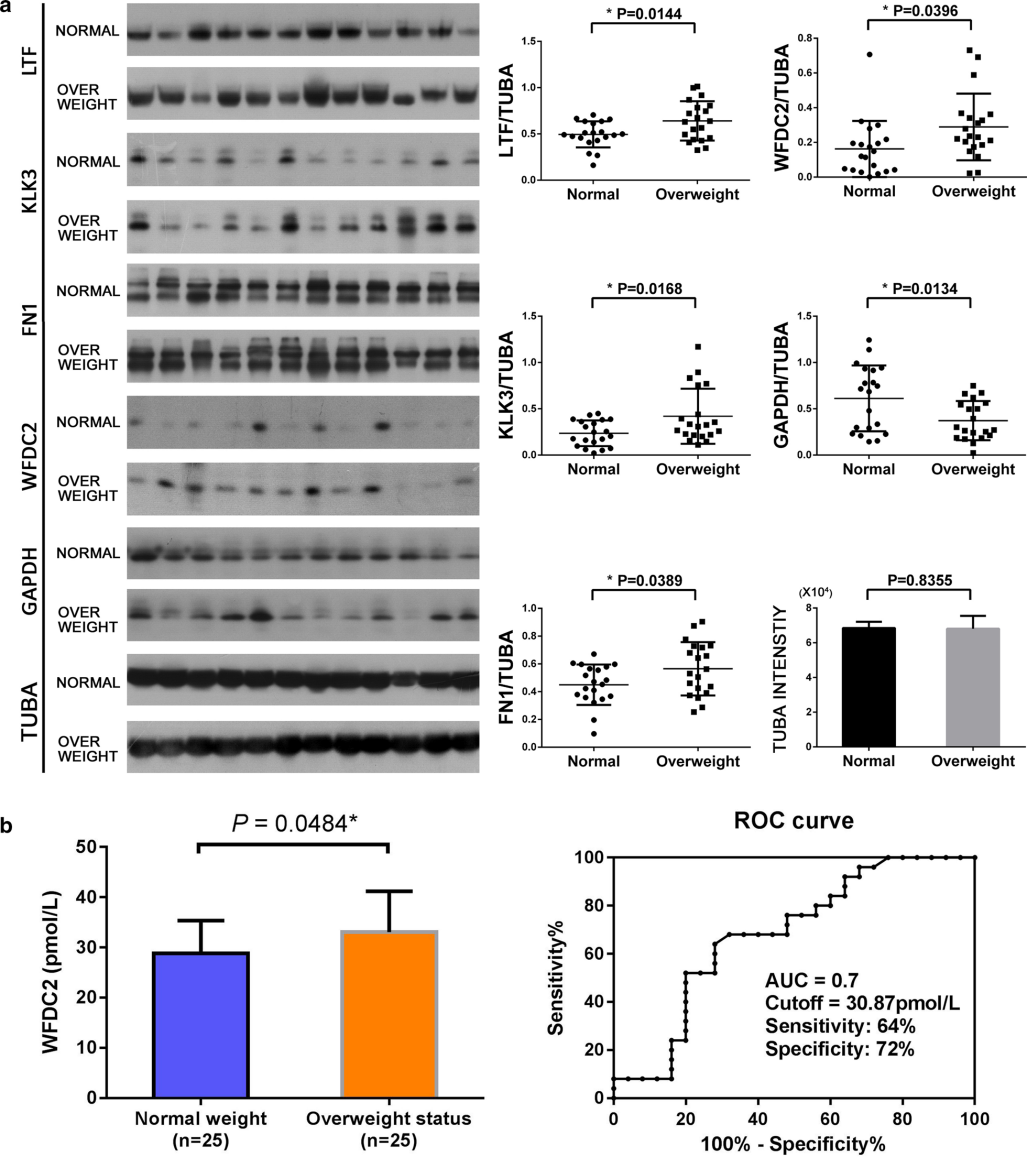
Representative western blot analyses of four altered HFF proteins and validation of the expression of upregulated protein WAP four-disulfide core domain protein 2 (WFDC2) by ELISA. **a** The expressions of WAP four-disulfide core domain protein 2 (WFDC2), lactotransferrin (LTF), Prostate-specific antigen (KLK3), Fibronectin (FN1), and glyceraldehyde 3-phosphate dehydrogenase (GAPDH) were confirmed by western blot. Asterisk denoted a statistically significant difference between the two groups; **a** WFDC2 concentration in the follicular fluid samples of 25 normal weight women and 25 overweight status women. ROC curve of WFDC2 distinguished the normal weight and overweight status women

To verify the confidence of the proteome data, the expression patterns of four HFF proteins (FN1, GAPDH, KLK3, and WFDC2) from 20 normal weight women and 20 overweight status women were analyzed by Western blot (Fig. 6a). The results indicated that the levels of the four proteins were consistent with the proteomic result. ELISA was used to detect the levels of WFDC2 in HFF of 25 normal weight women and 25 overweight status women. The result showed that WFDC2 protein level was elevated in the HFF of overweight status women (33.1 ± 1.6 pmol/L) compared with that of normal weight women (28.8 ± 1.3 pmol/L) (*p* = 0.0484). ROC curve analysis showed that the AUC was 0.7 to distinguish the overweight status women from the normal weight women, with a sensitivity of 64% and a specificity of 72% (Fig. 6b). This result indicated that WFDC2 could be a potential candidate HFF marker for the diagnosis of oocyte maturation arrest of the overweight status women with a relatively higher sensitivity.

## Discussion

Recently, the detrimental effect of overweight status on female reproductive health has received increasing attention, which is widely associated with anovulation, hormone disorders, conception delay, high miscarriage risk, and adverse IVF outcomes, and abnormal oocyte differentiation and maturation. In our previous retrospective cohort study of 411 non-PCOS female patients undergoing their first IVF-ET treatment, overweight status women had statistically significantly lower number of mature oocytes, fertilization rate, blast formation rate and embryo implantation rate than those of the normal-weight women [16]. Further, in order to better understand folliculogenesis in the ovary, we had applied a proteomic method to investigate the molecular compositions of HFF surrounding the ovum which provides a special milieu for the development of follicle and the maturation of oocytes, and then identified the HFF proteins associated with oocyte maturation and successful IVF outcome [14]. Therefore, we hypothesized that the alteration of HFF proteins of overweight status women might contribute to their poor oocyte quality. To avoid the influence of hormone disorders and subfertility caused by PCOS, the HFF samples from simple overweight status women without PCOS were recruited in this present work.

Ambekar et al. has used three different prefractionations coupled with LC–MS/MS to identify 480 HFF proteins with the FDR < 0.01 [12]. Recently, Lewandowska et al. has applied ultrafiltration, optional immunodepletion, and high pH RP-HPLC fractionation combined with SWATH-MS to explore the proteomic and peptidomic composition of HFF [13]. Compared with above two HFF proteomes, a total of 57% HFF proteins were identical to the previously reported pool of HFF proteins, which not only validated the good quality of their data, but also provide a new complementary dataset related to folliculogenesis and oocyte maturation to facilitate the integration of the HFF data generated from different MS platform in the future.

Follicle development has been considered controlled by inflammatory response [21]. In our previous report [14], 17 components involved in coagulation cascade and 21 components associated with complement cascade were identified, which were important for the oocyte maturation [21–23]. In the present study, complement component C9 and coagulation factor XII were down-regulated in the overweight status women. C9 has been considered involved in the inhibition of complement cascade activity in IVF-treated women with controlled ovarian stimulation [24]. Coagulation factor XII could activate the classical complement pathway through the cleavage of C1 [25]. Contrary to the above, Girardi considered that inhibition of complement activation could improve angiogenesis failure and rescue pregnancies [26]. How these cascade components affect the oocyte maturation remains to be further explored. Angiogenesis is an important process that provide enough nutrients to support follicle maturation and HFF formation [27]. Angiotensinogen and FN1 were both important angiogenic factors. The former was down-regulated, and the latter was up-regulated in the HFF from the overweight status women. This implicated angiogenesis deficiency in overweight women, which might lead to improper corpus luteum formation, and inadequate degradation of fibronectins causing the impediment of follicle wall expansion. Though KLK3 has kallikrein-related protease activity that catalyzes clot dissolution [28] and was up-regulated in the HFF from the overweight status women, it still could not efficiently degrade FN1 [29]. Compared with the HFF proteome of PCOS women [18], the alteration of HFF proteins associated with complement coagulation cascade and angiogenesis showed characteristic difference in the non-PCOS women. More researches are still needed in the future.

HFF-mediated communications between oocyte and follicular cells facilitate follicle development and oocyte maturation. In the process of follicle enlargement, the accumulation of laminin in the basal lamina is essential for the prediction of developmental competence of oocytes [30, 31]. Basement membrane-specific heparan sulfate proteoglycan core protein (HSPG2) is an integral component of basement membranes, and participates in the formation of basal lamina by intertwining with some extracellular matrix proteins. In the present work, HSPG2 was down-regulated and extracellular matrix protein 1 (ECM1) was up-regulated in overweight status women, indicating their potential adverse influence on normal follicle development.

WFDC2, also named human epididymis protein 4 (HE4) [32], has recently been found overexpressed in serous and endometrioid epithelial ovarian carcinomas [33]. Sandow et al. also suggested that WFDC2 might be a novel biomarker for ovarian cancer [34]. In the present work, WFDC2 was also found up-regulated in the HFF from the overweight status women. Meanwhile, ELISA assay indicated WFDC2 could distinguish the HFF of the normal-weight women from the HFF of the overweight status women with a relatively higher sensitivity. Therefore, WFDC2 might be a candidate HFF marker for the diagnosis of abnormal oocyte maturation in the overweight status women. Despite all this, more researches are still needed to further evaluate the diagnostic value of WFDC2.

In conclusion, the alteration of HFF proteins in overweight status women reflected the secretary function disorders of granulosa cells and thecae, and the breach of blood-follicle barrier, which were involved in abnormal folliculogenesis and oocyte maturation. Our work put new light on the better understanding of abnormal oocyte maturation caused by simple female overweight status without PCOS. Besides, due to the easy accessibility of HFF during the aspiration of oocytes in IVF treatment, it can be an ideal source for microinvasive screening of the oocyte maturation and other ovarian diseases.

## Supplementary information

**Additional file 1.** Additional table.

## Abbreviations

ICSI: Intracytoplasmic sperm injection
iTRAQ: Isobaric tags for relative and absolute quantification
HPLC: High-performance liquid chromatography
MALDI: Matrix-assisted laser desorption/ionization
MS: Mass spectrometry
WHO: World Health Organization
PCOS: Polycys-tic ovary syndrome
PANTHER: Protein analysis through evolutionary relationships
DAVID: The database for annotation, visualization and integrated discovery
STRING: Search tool for recurring instances of neighbouring genes
BMI: Body mass index
FDR: False discovery rate

## References

[1] Inhorn MC, Patrizio P (2015). Infertility around the globe: new thinking on gender, reproductive technologies and global movements in the 21st century. Hum Reprod Update..

[2] Ombelet W, Cooke I, Dyer S, Serour G, Devroey P (2008). Infertility and the provision of infertility medical services in developing countries. Hum Reprod Update..

[3] Rouchou B (2013). Consequences of infertility in developing countries. Perspect Public Health.

[4] Mascarenhas MN, Flaxman SR, Boerma T, Vanderpoel S, Steven GA (2012). National, regional, and global trends in infertility prevalence since 1990: a systematic analysis of 277 health surveys. PLoS Med..

[5] Silvestris E, de Pergola G, Rosania R, Loverro G (2018). Obesity as disruptor of the female fertility. Reprod Biol Endocrinol..

[6] Chavarro JE, Rich-Edwards JW, Rosner BA, Willett WC (2007). Diet and lifestyle in the prevention of ovulatory disorder infertility. Obstet Gynecol.

[7] Zamah AM, Hassis ME, Albertolle ME, Williams KE (2015). Proteomic analysis of human follicular fluid from fertile women. Clin Proteomics.

[8] Guo N, Liu P, Ding J, Zheng SJ, Yuan BF, Feng YQ (2016). Stable isotope labeling Liquid chromatography/mass spectrometry for quantitative analysis of androgenic and progestagenic steroids. Anal Chim Acta.

[9] Xia L, Zhao X, Sun Y, Hong Y, Gao Y, Hu S (2014). Metabolomic profiling of human follicular fluid from patients with repeated failure of in vitro fertilization using gas chromatography/mass spectrometry. Int J Clin Exp Pathol..

[10] Benkhalifa M, Madkour A, Louanjli N (2015). From global proteome profiling to single targeted molecules of follicular fluid and oocyte: contribution to embryo development and IVF outcome. Expert Rev Proteomics..

[11] Hashemitabar M, Bahmanzadeh M, Mostafaie A (2014). A proteomic analysis of human follicular fluid: comparison between younger and older women with normal FSH levels. Int J Mol Sci.

[12] Ambekar AS, Nirujogi RS, Srikanth SM, Chavan S, Kelkar DS, Hinduja I (2013). Proteomic analysis of human follicular fluid: a new perspective towards understanding folliculogenesis. J Proteomics..

[13] Lewandowska AE, Macur K, Czaplewska P, Liss J, Łukaszuk K, Ołdziej S (2018). Human follicular fluid proteomic and peptidomic composition quantitative studies by SWATH-MS methodology. Applicability of high pH RP-HPLC fractionation. J Proteomics..

[14] Shen X, Liu X, Zhu P (2017). Proteomic analysis of human follicular fluid associated with successful in vitro fertilization. Reprod Biol Endocrinol..

[15] NCD Risk Factor Collaboration (NCD-RisC) (1975). Worldwide trends in body-mass index, underweight, overweight, and obesity from to 2016: a pooled analysis of 2416 population-based measurement studies in 128·9 million children, adolescents, and adults. Lancet.

[16] Shen XF, Liu X, Zhang YH, Li N, Wang JH, Liu FJ (2016). Obesity impaired oocyte maturation and embryo implantation rate in Chinese women without polycystic ovary syndrome undergoing in vitro fertilization-embryo transfer. Int J Clin Exp Med..

[17] WHO (2000). Obesity: preventing and managing the global epidemic. Report of a WHO Consultation. WHO Technical Report Series 894.

[18] Ambekar AS, Kelkar DS, Pinto SM, Sharma R, Hinduja I, Zaveri K (2015). Proteomics of follicular fluid from women with polycystic ovary syndrome suggests molecular defects in follicular development. J Clin Endocrinol Metab.

[19] Gotham SM, Fryer PJ, Paterson WR (1998). The measurement of insoluble proteins using a modified Bradford assay. Anal Biochem.

[20] Sun W, Xing B, Guo L (2016). Quantitative proteomics analysis of tissue interstitial fluid for identification of novel serum candidate diagnostic marker for hepatocellular carcinoma. Sci Rep..

[21] Espey LL (1994). Current status of the hypothesis that mammalian ovulation is comparable to an inflammatory reaction. Biol Reprod.

[22] Ebisch IM, Thomas CM, Wetzels AM, Willemsen WN, Sweep FC, Steegers-Theunissen RP (2008). Review of the role of the plasminogen activator system and vascular endothelial growth factor in subfertility. Fertil Steril.

[23] Levi M, van der Poll T, Büller HR (2004). Bidirectional relation between inflammation and coagulation. Circulation.

[24] Jarkovska K, Martinkova J, Liskova L (2010). Proteome mining of human follicular fluid reveals a crucial role of complement cascade and key biological pathways in women undergoing in vitro fertilization. J Proteome Res.

[25] Ghebrehiwet B, Silverberg M, Kaplan AP (1981). Activation of the classical pathway of complement by Hageman factor fragment. J Exp Med.

[26] Girardi G (2008). Complement inhibition keeps mothers calm and avoids fetal rejection. Immunol Invest.

[27] Stouffer RL, Martínez-Chequer JC, Molskness TA, Xu F, Hazzard TM (2001). Regulation and action of angiogenic factors in the primate ovary. Arch Med Res.

[28] Mattsson JM, Ravela S, Hekim C (2014). Proteolytic activity of prostate-specific antigen (PSA) towards protein substrates and effect of peptides stimulating PSA activity. PLoS ONE.

[29] Lundwall A, Brattsand M (2008). Kallikrein-related peptidases. Cell Mol Life Sci.

[30] Rodgers RJ, Irving-Rodgers HF, Russell DL (2003). Extracellular matrix of the developing ovarian follicle. Reproduction.

[31] Irving-Rodgers HF, Morris S, Collett RA (2009). Phenotypes of the ovarian follicular basal lamina predict developmental competence of oocytes. Hum Reprod.

[32] Kirchhoff C (1998). Molecular characterization of epididymal proteins. Rev Reprod..

[33] Drapkin R, von Horsten HH, Lin Y (2005). Human epididymis protein 4 (HE4) is a secreted glycoprotein that is overexpressed by serous and endometrioid ovarian carcinomas. Cancer Res.

[34] Sandow JJ, Rainczuk A, Infusini G (2018). Discovery and validation of novel protein biomarkers in ovarian cancer patient urine. Proteomics Clin Appl..

